# The role of TyG index in predicting the incidence of diabetes in Chinese elderly men: a 20-year retrospective study

**DOI:** 10.3389/fendo.2023.1191090

**Published:** 2023-06-23

**Authors:** Lingjun Rong, Naijing Hou, Jingsheng Hu, Yanping Gong, Shuangtong Yan, Chunlin Li, Zaigang Yang, Banruo Sun

**Affiliations:** ^1^ Department of Geriatric Endocrinology, The First Affiliated Hospital of Zhengzhou University, Zhengzhou, China; ^2^ Department of Geriatric Endocrinology, The Second Medical Center and National Clinical Research Center for Geriatric Diseases, Chinese People's Liberation Army (PLA) General Hospital, Beijing, China; ^3^ Department of Health Care, The Second Medical Center and National Clinical Research Center for Geriatric Diseases, Chinese PLA General Hospital, Beijing, China

**Keywords:** triglyceride glucose index, prediction, type 2 diabetes mellitus, elderly male population, oral glucose tolerance test

## Abstract

**Background:**

The triglyceride glucose index (TyG index) has been regarded as a reliable surrogate marker of insulin resistance and an independent predictor of diabetes. However, few studies have reported the association between the TyG index and diabetes in the elderly population. Accordingly, this study aimed to investigate the association between the TyG index and diabetes progression in elderly Chinese.

**Methods:**

Baseline medical history, fasting plasma glucose (FPG), glucose levels during the oral glucose tolerance test (OGTT) after 1-hour (1h-PG) and 2-hour (2h-PG), and triglyceride (TG) were obtained from a cohort of 862 elderly (aged ≥ 60 years) Chinese in the Beijing urban area between 1998 and 1999. A follow-up visit was conducted between 1998 and 2019 to assess incident diabetes. TyG index was calculated by the following formula ln[TG (mg/dL) × FPG (mg(dL)/2]. The predictive values of TyG index, lipids, and glucose levels during OGTT were assessed alone and also in a clinical prediction model comprising traditional risk factors using concordance index (C-index). Areas under the receiver operating characteristics curves (AUC) and 95% CIs were calculated.

**Results:**

After 20 years of follow-up, there were 544 cases of incident type 2 diabetes mellitus (63.1% of incidence). The multivariable HRs (95% CI) for TyG index, FPG, 1h-PG and 2h-PG, high-density lipoprotein-cholesterol (HDL-c), and TG were 1.525 (1.290-1.804), 1.350 (1.181-1.544), 1.337 (1.282-1.395), 1.401 (1.327-1.480), 0.505 (0.375-0.681), and 1.120 (1.053-1.192), respectively. The corresponding C-index were 0.623, 0.617, 0.704, 0.694, 0.631, and 0.610, respectively. The AUC (95% CI) for the TyG index, FPG, 1h-PG, 2h-PG, HDL-c, and TG were 0.608 (0.569-0.647), 0.587 (0.548-0.625), 0.766 (0.734-0.797), 0.713 (0.679-0.747), 0.397 (0.358-0.435), and 0.588 (0.549-0.628). The AUC of the TyG index was higher than that of TG but did not differ with FPG and HDL-c. In addition, the AUCs of 1h-PG and 2h-PG were higher than that of the TyG index.

**Conclusions:**

Elevated TyG index is independently correlated with an increased risk of incident diabetes in the elderly male population, but it is not superior to OGTT 1h-PG and 2h-PG in predicting the risk of diabetes.

## Introduction

The International Diabetes Federation estimates that about 536.6 million people have diabetes worldwide, comprising 10.5% of the world’s population in 2021. The number is estimated to rise to 12.2% (783.2 million) in 2045 (1). According to the data of China’s seventh national population census, the elderly population (≥60 years old) accounted for 18.7% (260.4 million) of the total population in 2020. About 30% (78.13million) of them suffer from diabetes mellitus and more than 95% have type 2 diabetes mellitus (T2DM) (2). Age is a well-known strong risk factor for diabetes and cardiovascular diseases (CVD). T2DM can decrease life expectancy by as much as 10 years, and CVD are the leading causes of death among patients with T2DM (3). In addition, more than 45% of the elderly subjects are in pre-diabetes state (4). With the aging of the world population, many people develop asymptomatic hyperglycemia, which aggravates metabolic disorders, causes occult microvascular and macrovascular damage, and increases the risk of CVD. Due to the lack of sensitive predictors for the early detection of diabetes, irreversible vascular damage occurs in prediabetes or the early stage of diabetes. Accordingly, it is difficult to effectively reduce the incidence of cardiovascular complications through strict blood glucose control and other treatment methods at this time. Therefore, it is necessary to identify high-risk populations in the early stages. It allows the implementation of therapeutic strategies to delay the progression of diabetes and its complications.

Insulin resistance (IR) is a condition in which cells show diminished response to insulin regardless of hyperinsulinemia. IR is the major pathophysiological basis in the development of T2DM, and it may exist 10–20 years before diagnosis (5). Therefore, IR is a crucial indicator of impending diabetes. The hyperinsulinemic-euglycemic clamp technique, the gold-standard quantitative technique for measuring IR, is costly and time-consuming. Therefore, markers of IR which are simple and easy to obtain are badly needed. Evidence indicates that the visceral adiposity indexes such as body mass index (BMI) and waist circumference (WC) have the ability to identify IR. Meanwhile, there is a accumulating evidence that glucose levels during the oral glucose tolerance test (OGTT) after 1-hour (1h-PG) and 2-hour (2h-PG) are effective indicators for predicting T2DM (6–8). Furthermore, dyslipidemia is a major risk factor for T2DM and CVD. There is evidence suggesting that traditional lipid index, such as total cholesterol (TC), triglyceride (TG), high-density lipoprotein cholesterol (HDL-c) and Low-density lipoprotein cholesterol (LDL-c), are effective in detecting IR. Recently, the triglyceride-glucose index (TyG index), derived from TG and fasting plasma glucose (FPG), has been recommended as an alternative and ideal indicator instead of conventional insulin-based IR indices (9). Several studies demonstrated that the TyG index has a higher predictive value for diagnosing diabetes in Asian populations, especially in Chinese (10–14). However, most studies are inconsistent and support the relationship between the TyG index and T2DM risk in young and middle-aged individuals. The relationship between the TyG index and the risk of diabetes among elderly populations remains unclear.

Therefore, our study aimed to explore the association between the TyG index and incident diabetes and to assess its superiority over other traditional risk factors, particularly over glucose values obtained from oral glucose tolerance test which is widely used to diagnose diabetes in China. Thus, our study aimed to identify a better predictor for incident diabetes in the Chinese elderly male population.

## Materials and methods

### Study subjects

**Figure 1:**
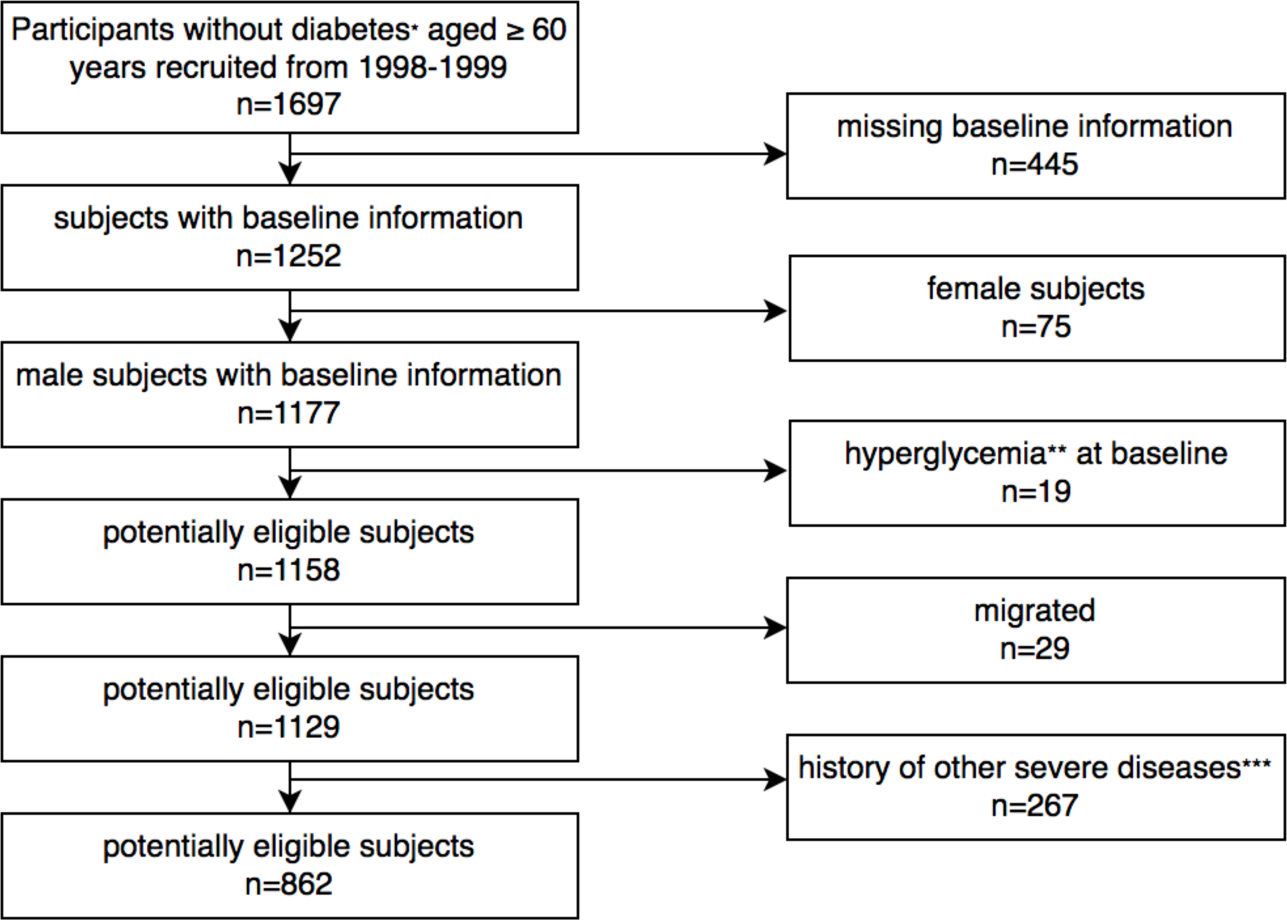
Flowchart showing study population selection. *According to the WHO criteria published in 1999. ** Secondary hyperglycemia caused by hypercortisolism, hypothyroidism, acromegaly, long-term use of steroids and other diseases. ***Including anemia, malignant tumor, severe liver, kidney, or gastrointestinal disease.

The present study was conducted at the Chinese PLA General Hospital of Beijing between May 1998 and August 2019. Patients aged 60 years or older were enrolled during their visits to outpatient clinics. All essential clinical data were recorded, including patient demographics, diagnoses, their main underlying diseases, and laboratory results. The subjects were then re-examined every one to two years during the follow-up period. Their laboratory results, hospitalization, and visits to outpatient clinics and emergency departments were also recorded. In total, 1697 subjects signed the informed consent form and were enrolled in this study. Of them, 862 male subjects aged ≥60 years without diabetes were included in the final analysis (**Figure 1**). The exclusion criteria were (1) Severe liver, kidney, or gastrointestinal disease (2) Anemia (3) Malignant tumor (4) Long-term use of steroids (5) Secondary hyperglycemia caused by hypercortisolism, hypothyroidism, acromegaly, and other diseases. The study protocol was approved by the Chinese PLA General Hospital Ethics Committee.

### Clinical data and biochemical indicators

Anthropometric measurements, including the determination of height, weight, waist circumference (WC), and blood pressure, were performed by a trained nurse according to standardized methods. Weight was quantified with subjects wearing light clothing and to the nearest 0.1 kg. Height was measured without shoes to the nearest 0.01 m. WC was measured to an accuracy of 0.01 m by the same staff. Body mass index (BMI) was calculated as weight divided by the square of the body height and expressed in kg/m^2^. Before blood pressure measurement, the subjects were seated for at least 5 minutes. The measurements were performed three times with an interval of 1 minute using an OMRON electronic blood pressure device and the average systolic blood pressure (SBP) and diastolic blood pressure (DBP) were recorded. Routine biochemical data, including TG, total cholesterol (TC), and high-density lipoprotein cholesterol (HDL-c), were also retrieved. Low-density lipoprotein cholesterol (LDL-c) level was computed with the Friedewald equation. Blood samples were drawn after 8-h of fasting and measured by chemiluminescence on an autoanalyzer under strict quality control. All subjects underwent 75 g oral glucose tolerance test (OGTT) and venous plasma glucose measurements were taken before OGTT (FPG), and 1-hour and 2-hour after OGTT (1h-PG and 2h-PG, respectively). The enzymatic hexokinase method was used to measure FPG, 1h-PG, and 2h-PG. The TyG index was calculated by the following formula ln[TG (mg/dl) × FPG (mg(dl)/2].

### Definition of variables and T2DM

Based on the Chinese guideline for the management of dyslipidemia in adults (revised in 2016) (15), the groups of lipid parameters were as follows: TG: normal: < 1.7 mmol/L, borderline high: 1.7–2.3 mmol/L, high: ≥ 2.3 mmol/L; TC: normal: < 5.2 mmol/L, borderline high: 5.2–6.2 mmol/L, high: ≥ 6.2 mmol/L; HDL-c: low risk: ≥ 1.0 mmol/L, high risk: < 1.0 mmol/L; LDL-c: normal < 3.4 mmol/L, borderline high: 3.4–4.1 mmol/, high: ≥ 4.1 mmol/L. Hypertension was defined as the presence of one of the following conditions: SBP ≥ 140 mmHg or DBP ≥ 90 mmH or taking antihypertensive medications due to the previous diagnosis by clinicians. The diagnosis of T2DM was defined as the primary end-point of the study. We diagnosed diabetes according to the WHO criteria published in 1999: symptoms of diabetes plus random plasma glucose concentration ≥ 200 mg/dL (11.1 mmol/L), or FPG ≥ 126 mg/dL (7.0 mmol/L), or 2h-PG ≥ 200 mg/dL (11.1 mmol/L) during an OGTT. Glucose tolerance was determined according to the WHO criteria in 1999 as normal glucose tolerance (FPG ≤6 mmol/L and 2h-PG <7.8 mmol/L) and prediabetes (including impaired fasting glucose (IFG) (6 mmol/L< FPG <7 mmol/L and 2h-PG <7.8 mmol/L) and impaired glucose tolerance (IGT) (FPG <7mmol/L and 7.8 mmol/L≤ 2h-PG <11.1 mmol/L)). The TyG index and 1h-PG were divided into three groups. FPG and 2h-PG were divided as follows: FPG: normal: ≤6 mmol/L, IFG: >6 mmol/L; 2h-PG: normal: <7.8 mmol/L, IGT: ≥7.8 mmol/L. The follow-up period for each subject was defined as the elapsed time from the baseline screening to T2DM diagnosis, death, emigration, or the last follow-up date of up to 20 years.

### Statistical analysis

Continuous variables are expressed as the mean ± standard error (SE) or interquartile range for variables with normal and skewed distribution, respectively. Categorical variables are presented as frequencies and percentages. The subjects were classified based on the development of T2DM. Differences in the two groups were examined by Student’s t test, Mann–Whitney U test, and chi-squared test, as appropriate. To define potential indicators of T2DM, univariable Cox proportional-hazards regressions were conducted on the following demographic and clinical variables: age, BMI, WC, SBP, DBP, and history of hypertension. Statistically significant and clinically relevant variables were included in the multivariable Cox models. The predictive value, both alone and in addition to the clinical prediction model, was tested with concordance index (C-index). The area under the curve (AUC) of the receiver operating characteristics (ROC) plot and the 95% CI were calculated to compare the predictive power of each risk factor. All statistical analyses were performed using SPSS version 25 and R Version 4.0.2. A two-tailed p-value of < 0.05 was considered statistically significant.

## Results

### Baseline characteristics of the subjects

The study included 862 subjects with a median (25^th^–75^th^ percentile) age at baseline of 74.0(68.0-79.0) years who were followed for 20 years. At baseline, 491 subjects had normal NGT, 53 had IFG, and 318 had IGT. During 9982 person-years of follow-up, there were 544 incident cases of T2DM (overall incidence of 54.5 cases/1000 person-years). Incidence was 33.8 cases/1000 person-years for NGT and 102.5 cases/1000 person-years for prediabetes (**Table 1**). **Table 2** presents the anthropometric and metabolic characteristics of the participants. Subjects who progressed to T2DM were older and had higher BMI, WC, TG, SBP, FPG, 1h-PG, 2h-PG, and TyG index. HDL-c was lower in subjects who progressed to T2DM compared with subjects who remained non-diabetic during follow-up. As shown in **Table 3**, the population was categorized based on the TyG index tertiles. The participants in the highest tertile of the TyG index had generally worse risk profile than the other tertiles of the TyG index, while the subjects in the lowest tertile of TyG index were older.

**Table 1:** Incidence rate for the development of T2DM by TyG index and glucose tolerance.

	Total (n=862)	TyG index			Glucose tolerance	
		T1 (n=284)	T2 (n=293)	T3 (n=285)	NGT (n=491)	Prediabetes (n=371)
No. of cases	544	148	190	206	236	308
No. of person-years	9982	3753	3259	2970	6976	3006
Incidence rate*	54.5	39.4	58.3	69.4	33.8	102.5

*per 1000 person-years.

**Table 2:** Baseline characteristics stratified by development of T2DM.

Variables	Total (n=862)	Development of T2DM		P
		No (n=318)	Yes (n=544)	
Age (years)	74.0 (68.0-79.0)	73.0 (67.0-78.0)	75.0 (68.0-79.0)	0.001
BMI (kg/m^2^)	25.2 ± 0.10	24.8 ± 0.15	25.5 ± 0.12	0.001
WC (cm)	89.4 ± 0.30	87.8 ± 0.48	90.4 ± 0.37	<0.001
SBP (mmHg)	130 (120-140)	130 (120-140)	135 (120-145)	0.001
DBP (mmHg)	75 (70-80)	75 (70-80)	76 (70-80)	0.222
HDL-c (mmol/l)	1.2 ± 0.01	1.3 ± 0.02	1.2 ± 0.02	<0.001
LDL-c (mmol/l)	3.2 ± 0.03	3.2 ± 0.05	3.3 ± 0.04	0.439
TC (mmol/l)	5.3 ± 0.03	5.2 ± 0.05	5.3 ± 0.04	0.386
TG (mmol/l)	1.8 ± 0.04	1.6 ± 0.04	1.9 ± 0.05	<0.001
TyG index	8.7 ± 0.02	8.6 ± 0.03	8.8 ± 0.02	<0.001
FPG (mmol/l)	4.9 ± 0.02	4.8 ± 0.03	5.0 ± 0.03	<0.001
1h-PG (mmol/l)	9.5 ± 0.07	8.4 ± 0.09	10.2 ± 0.08	<0.001
2h-PG (mmol/l)	7.4 ± 0.06	6.6 ± 0.08	7.9 ± 0.08	<0.001
Hypertension (n,%)	518 (60.1)	170 (19.7)	348 (40.4)	0.002

Data were mean ± SE or median (IQR) for skewed variables or numbers (proportions) for categorical variables.

T2DM, type 2 diabetes mellitus; BMI, body mass index; WC, waist circumference; SBP, systolic blood pressure; DBP, diastolic blood pressure; HDL-c, high-density lipoprotein cholesterol; LDL-c, low-density lipoprotein cholesterol; TC, total cholesterol; TG, triglyceride; TyG index, triglyceride fasting glucose index; FPG, fasting plasma glucose; 1h-PG, 1-hour plasma glucose concentration; 2h-PG, 2-hour plasma glucose concentration.

**Table 3:** Baseline characteristics stratified by tertiles of TyG index.

Variables	Tertile 1 n=284	Tertile 2 n=293	Tertile 3 n=285	P
Age (years)	75.0 (69.0-80.0)	74.0 (68.0-78.5)	73.0 (67.0-78.0)	0.019
BMI (kg/m^2^)	24.7 ± 0.17	25.3 ± 0.16	25.7 ± 0.16	<0.001
WC (cm)	88.3 ± 0.54	89.2 ± 0.50	90.7 ± 0.51	0.005
SBP (mmHg)	130 (120-140)	130 (120-140)	130 (120-140)	0.831
DBP (mmHg)	75 (70-80)	75 (70-80)	80 (70-82)	0.020
HDL-c (mmol/l)	1.3 ± 0.02	1.2 ± 0.02	1.2 ± 0.03	<0.001
LDL-c (mmol/l)	3.2 ± 0.05	3.4 ± 0.05	3.1 ± 0.06	0.001
TC (mmol/l)	5.0 ± 0.05	5.3 ± 0.05	5.5 ± 0.06	<0.001
TG (mmol/l)	1.1 ± 0.02	1.6 ± 0.01	2.7 ± 0.09	<0.001
TyG index	8.2 ± 0.02	8.7 ± 0.01	9.2 ± 0.02	<0.001
FPG (mmol/l)	4.6 ± 0.04	4.9 ± 0.03	5.2 ± 0.04	<0.001
1h-PG (mmol/l)	9.2 ± 0.11	9.5 ± 0.11	10.0 ± 0.13	<0.001
2h-PG (mmol/l)	7.0 ± 0.10	7.5 ± 0.10	7.8 ± 0.10	<0.001
Hypertension (n,%)	167 (19.4)	176 (20.4)	175 (20.3)	0.818

Data were mean ± SE or median (IQR) for skewed variables or numbers (proportions) for categorical variables.

T2DM, type 2 diabetes mellitus; BMI, body mass index; WC, waist circumference; SBP, systolic blood pressure; DBP, diastolic blood pressure; HDL-c, high-density lipoprotein cholesterol; LDL-c, low-density lipoprotein cholesterol; TC, total cholesterol; TG, triglyceride; TyG index, triglyceride fasting glucose index; FPG, fasting plasma glucose; 1h-PG, 1-hour plasma glucose concentration; 2h-PG, 2-hour plasma glucose concentration.

### The TyG index was a predictive value for incident diabetes

According to **Table 4**, as categorical variables, changes in the TyG index and 1h-PG levels from the first to the third tertiles showed significant increasing trends for incident T2DM. The HR of incident T2DM increased with higher LDL-c (≥3.4mmol/L), higher TG (≥1.7mmol/L), higher FPG (>6.0mmol/L), higher 2h-PG (≥7.8mmol/L), or lower HDL-c (<1.0mmol/L). This association persisted even after adjusting for age, BMI, WC, SBP, and hypertension. The risk of incident T2DM increased with increasing tertiles of the TyG index. Compared with the first tertile, the corresponding HRs are as follows: tertile 2, 1.457 (95%CI 1.174-1.808) and tertile 3, 1.704 (95%CI 1.375-2.111). However, higher TC (≥5.2mmol/L) was not associated with incident T2DM.

**Table 4:** Multivariate-adjusted HRs (95%CI) of HDL-c, LDL-c, TG, TyG index, FPG, 1h-PG and 2h-PG for incidence of T2DM at 20 years of follow-up.

Variables	Non-adjusted HR (95% CI)^a^	P	Age-adjusted HR (95% CI)^a^	P	Multivariate^b^ HR (95% CI)^a^	P
HDL-c, mmol/L						
≥1.0	1		1		1	
<1.0	1.462 (1.197-1.786)	<0.001	1.410 (1.154-1.723)	0.001	1.421 (1.163-1.737)	0.001
LDL-c, mmol/L						
<3.4	1		1		1	
≥3.4	1.217 (1.028-1.441)	0.023	1.201 (1.015-1.422)	0.033	1.232 (1.040-1.459)	0.016
TG, mmol/L						
<1.7	1		1			
≥1.7	1.411 (1.192-1.670)	<0.001	1.459 (1.232-1.728)	<0.001	1.400 (1.180-1.660)	<0.001
TyG index						
T1	1		1		1	
T2	1.451 (1.170-1.799)	0.001	1.503 (1.212-1.864)	<0.001	1.457 (1.174-1.808)	0.001
T3	1.702 (1.377-2.103)	<0.001	1.800 (1.455-2.227)	<0.001	1.704 (1.375-2.111)	<0.001
FPG, mmol/L						
≤6	1		1		1	
>6	2.105 (1.505-2.943)	<0.001	2.152 (1.539-3.009)	<0.001	2.036 (1.454-2.851)	<0.001
1h-PG						
T1	1		1		1	
T2	1.821 (1.436-2.310)	<0.001	1.798 (1.417-2.280)	<0.001	1.802 (1.421-2.285)	<0.001
T3	4.335 (3.464-5.425)	<0.001	4.242 (3.388-5.311)	<0.001	4.150 (3.314-5.199)	<0.001
2h-PG, mmol/L						
<7.8	1		1		1	
≥7.8	2.840 (2.394-3.370)	<0.001	2.747 (2.313-3.264)	<0.001	2.754 (2.318-3.273)	<0.001

HR, hazard ratio; CI, confidence interval; HDL-c, high-density lipoprotein cholesterol; LDL-c, low-density lipoprotein cholesterol; TG, triglyceride; TyG index, triglyceride fasting glucose index; FPG, fasting plasma glucose; 1h-PG, 1-hour plasma glucose concentration; 2h-PG, 2-hour plasma glucose concentration.

a HR=1 as reference level.

b variables were age, BMI, WC, SBP and hypertension.

**Table 5** shows the association between 1-SD increase in lipids, plasma glucose and TyG index, and incident T2DM based on univariable and multivariable Cox proportional hazard analyses. After adjusting for age, BMI, WC, SBP, and hypertension, 1-SD increase in TyG index, FPG, 1h-PG, 2h-PG, HDL-c or TG for incident T2DM were 1.525 (95%CI 1.290-1.804), 1.350 (95%CI 1.181-1.544), 1.337 (95%CI 1.282-1.395), 1.401 (95%CI 1.327-1.480), 0.505 (95%CI 0.375-0.681), and 1.120 (95%CI 1.053-1.192), respectively. LDL-c and TC did not predict the incidence of T2DM. The C-index for TyG index alone was greater than TG, but did not significantly differ with PFG and HDL-c. The C-index for 1h-PG and 2h-PG was greater than that of the TyG index. To compare the predictive value of lipids, plasma glucose, and TyG index in the conventional model, including age, BMI, WC, SBP, and hypertension, multivariable Cox proportional hazard analyses and C-index were conducted. The C-index did not improve after adding the TyG index to the conventional model compared to HDL-c and TG. Furthermore, after adding to the conventional model, the C-index of 1h-PG (0.704) and 2h-PG (0.694) were greater than that of the TyG index (0.623).

**Table 5:** Multivariate-adjusted HRs (95%CI) and C-index of TyG index, FPG, 1h-PG, 2h-PG, HDL-c, LDL-c, TC and TG for incidence of T2DM at 20 years of follow-up.

Variables	Non-adjusted HR (95% CI)	P	C-index	P^a^	Multivariate ^b^ HR (95% CI)	P	C-index	P^a^
TyG index	1.538 (1.307-1.810)	<0.001	0.570	Ref.	1.525 (1.290-1.804)	<0.001	0.623	Ref.
FPG	1.366 (1.193-1.563)	<0.001	0.557	0.366	1.350 (1.181-1.544)	<0.001	0.617	0.591
1h-PG	1.364 (1.309-1.420)	<0.001	0.688	<0.001	1.337 (1.282-1.395)	<0.001	0.704	0.001
2h-PG	1.452 (1.375-1.533)	<0.001	0.680	<0.001	1.401 (1.327-1.480)	<0.001	0.694	<0.001
HDL-c	0.453 (0.338-0.609)	<0.001	0.594	0.175	0.505 (0.375-0.681)	<0.001	0.631	0.106
LDL-c	1.036 (0.941-1.141)	0.490	–	–	1.049 (0.951-1.156)	0.341	–	–
TC	1.015 (0.924-1.114)	0.778	–	–	1.034 (0.942-1.136)	0.479	–	–
TG	1.126 (1.060-1.196)	<0.001	0.558	0.005	1.120 (1.053-1.192)	<0.001	0.610	0.125

HR, hazard ratio; CI, confidence interval; TyG index, triglyceride fasting glucose index; FPG, fasting plasma glucose; 1h-PG, 1-hour plasma glucose concentration; 2h-PG, 2-hour plasma glucose concentration; HDL-c, high-density lipoprotein cholesterol; LDL-c, low-density lipoprotein cholesterol; TC, total cholesterol; TG, triglyceride.

a P value are the comparisons of C-index for the model including TyG index vs C-index for models including other variables.

b Included variables were age, BMI, WC, SBP and hypertension.

-, not applicable.

### The TyG index is not superior to 1h-OGTT PG and 2h-OGTT PG in predicting the incidence of diabetes.

To examine whether the TyG index is a better indicator for incident T2DM, the predictive values of the variables in a ROC analysis were compared. The AUC (95% CI) of the TyG index for T2DM, 0.608 (0.569-0.647), was greater than that of TG. In contrast, the AUCs (95% CI) of 1h-PG 0.766 (0.734-0.797) and 2h-PG 0.713 (0.679-0.747) were greater than that of TyG index (**Table 6**).

**Table 6:** AUC for each evaluated variable in predicting T2DM.

Variables	AUC (95% CI)	P
TyG index	0.608 (0.569-0.647)	Ref.
FPG	0.587 (0.548-0.625)	0.348
1h-PG	0.766 (0.734-0.797)	<0.001
2h-PG	0.713 (0.679-0.747)	<0.001
age	0.570 (0.531-0.609)	0.206
BMI	0.569 (0.530-0.608)	0.126
WC	0.588 (0.548-0.627)	0.443
TG	0.588 (0.549-0.628)	0.003
TC	0.530 (0.490-0.570)	–
HDL-c	0.397 (0.358-0.435)	0.859
LDL-c	0.535 (0.495-0.574)	–
SBP	0.569 (0.529-0.608)	0.164

AUC, area under the curve; CI, confidence interval; TyG index, triglyceride fasting glucose index; FPG, fasting plasma glucose; 1h-PG, 1-hour plasma glucose concentration; 2h-PG, 2-hour plasma glucose concentration; BMI, body mass index; WC, waist circumference; TG, triglyceride; TC, total cholesterol; HDL-c, high-density lipoprotein cholesterol; LDL-c, low-density lipoprotein cholesterol; SBP, systolic blood pressure.

P value from the comparisons of AUCs, the reference indicator was TyG index.

-, not applicable.

## Discussion

This population-based study demonstrated a longitudinal relationship between the TyG index and the risk of incident diabetes during a 20-year period follow-up of older Chinese adults in urban area. We examined the impact of lipids, plasma glucose, and TyG index on incident T2DM. We found that HDL-c, TG, TyG index, FPG, 1h-OGTT PG, and 2h-OGTT were significantly associated with incident T2DM, independent of major conventional risk factors. However, LDL-c and TC could not significantly predict the incidence of T2DM. Based on C-index and AUC, the strong predictors were OGTT 1h-PG and 2h-PG, and the TyG index was not superior to PFG and HDL-c.

Prospective studies have indicated that IR is the main etiology of diabetes and establishes many years before diagnosis (16). Previous studies found that the TyG index is more suitable for determining IR than alternative indexes, such as the homeostasis model assessment of insulin resistance (HOMA-IR) (17). The association between the TyG index and incident T2DM was also examined in China, South Korea, and other countries (12, 13, 18, 19). A study conducted in Singapore, showed that after adjusting for potential confounders, TyG accounted for 35.1% of the association between BMI and T2DM development (12). A Chinese cohort in the rural population revealed an increased risk of T2DM with an increasing TyG index for normal-weight people. Although the AUC of the TyG index was not better than WC or waist-to-height ratio among men (13). Another Chinese cohort showed that an elevated TyG index is independently correlated with a greater risk of incident diabetes, and the positive association is stronger among subjects with less than 40 years of age (10). In a recent community-based study conducted among Korean subjects with a mean age of 51.5 years and normal weight, it was shown that the AUC of the TyG index was significantly higher than that of IFG and similar to that of IGT (19). Furthermore, in another longitudinal study in China, a multivariate logistic regression model was used to analyze the relationship between baseline obesity, lipid levels, and non-insulin resistance index and the incidence of prediabetes in 4,543 subjects during 3.25 years. The study indicated that the TyG index may be a potential predictor of prediabetes in high-risk individuals (20). Similarly, our results showed that the TyG index predicts future T2DM.

The elderly population (≥60 years old) made up to 17.3 percent (240 million) of China’s total population in 2017, and the proportion is expected to exceed 30 percent by 2050, according to data released by the National Bureau of Statistics in 2018. More and more studies identified a significant relationship between age and CVD, and male patients with T2DM had higher rates of prevalent disease than females (3, 21). Although the TyG index is widely accepted as an easily measured and highly accurate predictor of diabetes. It remains unclear whether the TyG index is superior to FPG, 1h-OGTT PG, 2h-OGTT PG, lipid profile, and other indices for predicting the incidence of diabetes in older individuals. However, our findings revealed that the TyG index did not have an advantage over other lipid markers in predicting T2DM. Even it was inferior to postprandial blood glucose in elderly male population. The TyG index was previously introduced as a surrogate marker of IR (22, 23). Fernando et al. compared the TyG index with the euglycemic‐hyperinsulinemic clamp test, and proposed that the TyG index could be used to identify individuals with decreased insulin sensitivity (22). IR is largely due to impaired absorption of insulin-stimulated glucose in the skeletal muscle. A significant increase in TG levels in the peripheral blood and skeletal muscle impairs glucose metabolism in skeletal muscle (24). Therefore, the TyG index reflects muscle IR to a certain extent (25). Studies found that IR is not an inherent feature of older age. Obesity and physical inactivity are responsible for the purported IR in aging (26, 27). After accounting for both obesity and physical activity, aging per se is not associated with IR (26). Although insulin resistance contributes to altered glucose homeostasis, current evidence shows that the direct effect of aging on diabetes is exerted through the impairment function of β-cell, resulting in decreased insulin secretion (28). In the setting of genetic and lifestyle-related risk factors, aging contributes to the development of T2DM through impaired β-cell function and impaired β-cell adaptation (29), leading to impaired insulin secretion (30). A 50% reduction in β-cell secretory capacity has been observed in older men compared with younger men in response to arginine stimulation (31).

Among elderly subjects in China, increased postprandial blood glucose is the most common type of diabetes. Even when combined with FPG and glycosylated hemoglobin (HbA1c) for screening, 1/3 of patients with postprandial hyperglycemia are not diagnosed. Therefore, postprandial blood glucose is crucial for identifying early perturbation of glucose metabolism among elderly subjects. Several studies showed that compared to FPG, the glucose tolerance test is more sensitive for identifying individuals at high risk of prediabetes and diabetes in Asian populations, especially among older adults (32, 33). Studies based on Chinese population suggest that 2h-PG independently predicts the outcomes in models, including FPG and HbA1c. Therefore, in addition to FPG and HbA1c measurements, 2h-PG should be routinely measured to better assess the risks of diabetes and its complications (34). Post-load hyperglycemia differs from fasting hyperglycemia regarding its pathophysiology and clinical outcomes. First phase insulin secretion and hepatic insulin sensitivity are important determinants of the initial rise in plasma glucose concentration after glucose ingestion (35). The decline rate of plasma glucose concentration depends on late-phase insulin secretion and insulin sensitivity in the skeletal muscle (35). Thus, post-load plasma glucose can indirectly reflect β cell function and insulin sensitivity. However, fasting TG and FPG does not provide metabolically relevant information. Thus, the TyG index cannot be used instead of post-load plasma glucose for predicting diabetes in older populations.

Shorter examination time and lower cost are associated with greater convenience for both patients and physicians. We verified that the TyG index could be a screening tool to use for predicting future T2DM in Chinese elderly male population since that TyG index is a simple and low-cost biomarker often used in clinical practice. Therefore, the early detection of the TyG index may be beneficial for early interventions to prevent diabetes among the elderly patients. Notably, our data also provide evidence that TyG index is a less efficient predictor than 1h-PG and 2h-PG of incident T2DM, which mainly due to the special characteristics in elderly. Consequently, the elderly individuals at high risk for T2DM may be identified earlier by using the 1h-PG and 2h-PG as more reliable predictors to further delay the development of T2DM and its complication than TyG index.

There are, however, several limitations of this study. All subjects in this study were male in the urban area of Beijing, and the sample does not fully represent all Chinese elderly. This study included quite a large number of elderly Chinese, but we only enrolled subjects with better compliance with annual follow-ups and check-ups. It may bias our primary findings. We did not have data on the family history of diabetes. All subjects in our study were elderly; most of their parents barely knew about their health status and died long ago. Therefore, it is difficult to obtain an accurate family history of diabetes for all participants.

## Conclusion

The TyG index is an independent predictor of diabetes but it is not superior to OGTT 1h-PG and 2h-PG for predicting the incidence of diabetes among the elderly male population. We propose that OGTT, which is widely used to diagnose diabetes in China, is better than the TyG index for identifying metabolically unhealthy individuals and who have high risk of diabetes among older Chinese adults.

## Data availability statement

The raw data supporting the conclusions of this article will be made available by the authors, without undue reservation.

## Ethics statement

The studies involving human participants were reviewed and approved by Chinese PLA General Hospital Ethics Committee. The patients/participants provided their written informed consent to participate in this study.

## Author contributions

BS and ZY had full access to all the data in the study and designed the study. LR analyzed data and drafted the manuscript. NH, JH, YG, SY, and CL collected the data. BS revised the manuscript. All authors contributed to the article and approved the submitted version.
